# Cytokine-induced memory-like NK cells combined with Tafasitamab demonstrate efficacy against B-cell acute lymphoblastic leukemia

**DOI:** 10.1093/immadv/ltaf025

**Published:** 2025-07-16

**Authors:** Dimitrios Filioglou, Geovana S F Leite, Helena Batatinha, Nina Santa-Cruz, Dan W Davini, Forrest L Baker, Richard J Simpson, Emmanuel Katsanis

**Affiliations:** Department of Pediatrics, University of Arizona, Tucson, AZ, United States; Department of Pediatrics, University of Arizona, Tucson, AZ, United States; School of Nutritional Sciences and Wellness, University of Arizona, Tucson, AZ, United States; Department of Pediatrics, University of Arizona, Tucson, AZ, United States; Department of Pediatrics, University of Arizona, Tucson, AZ, United States; Department of Pediatrics, University of Arizona, Tucson, AZ, United States; School of Nutritional Sciences and Wellness, University of Arizona, Tucson, AZ, United States; University of Arizona Cancer Center, The University of Arizona, Tucson, AZ, United States; Department of Pediatrics, University of Arizona, Tucson, AZ, United States; School of Nutritional Sciences and Wellness, University of Arizona, Tucson, AZ, United States; University of Arizona Cancer Center, The University of Arizona, Tucson, AZ, United States; Department of Immunobiology, The University of Arizona, Tucson, AZ, United States; Department of Pediatrics, University of Arizona, Tucson, AZ, United States; University of Arizona Cancer Center, The University of Arizona, Tucson, AZ, United States; Department of Immunobiology, The University of Arizona, Tucson, AZ, United States

**Keywords:** combination immunotherapy, B-cell acute lymphoblastic leukemia, cytokine-induced memory-like NK cells, tafasitamab

## Abstract

Cytokine-induced memory-like natural killer cells (CIMLNK) represent a novel form of adoptive cellular therapy that is easy to manufacture and readily available. These cells are generated after overnight stimulation of purified natural killer (NK) cells with interleukin-12 (IL-12), interleukin-15 (IL-15), and interleukin-18 (IL-18). While CIMLNK has demonstrated efficacy in patients with relapsed or refractory acute myeloid leukemia (AML), its potential application in B-cell acute lymphoblastic leukemia (B-ALL) remains unclear. Tafasitamab (TAFA), a monoclonal antibody (mAb) directed against CD19, a surface antigen expressed on B-ALL cells, has been developed to augment anti-tumor efficacy through antibody-dependent cellular cytotoxicity (ADCC), a mechanism predominantly mediated by NK cells. Consequently, we sought to assess the susceptibility of B-ALL to the combination of CIMLNK and TAFA using three B-ALL cell lines: NALM6, SUP-B15, and RS4;11. The addition of TAFA significantly augmented the cytotoxic activity, degranulation capacity, and IFN-γ production of CIMLNK. TAFA-induced ADCC was found to be dose-dependent and was abolished after CD16 blockade. Furthermore, TAFA-mediated effects against NALM6 and SUP-B15 were more pronounced in CIMLNK compared to unstimulated NK cells. In vivo, the combination of CIMLNK and TAFA led to a more pronounced survival benefit in leukemia-bearing mice. In summary, our findings suggest that this combination holds promise as a potential alternative treatment option for patients with relapsed refractory B-ALL.

## Graphical Abstract

**Graphical Abstract:**
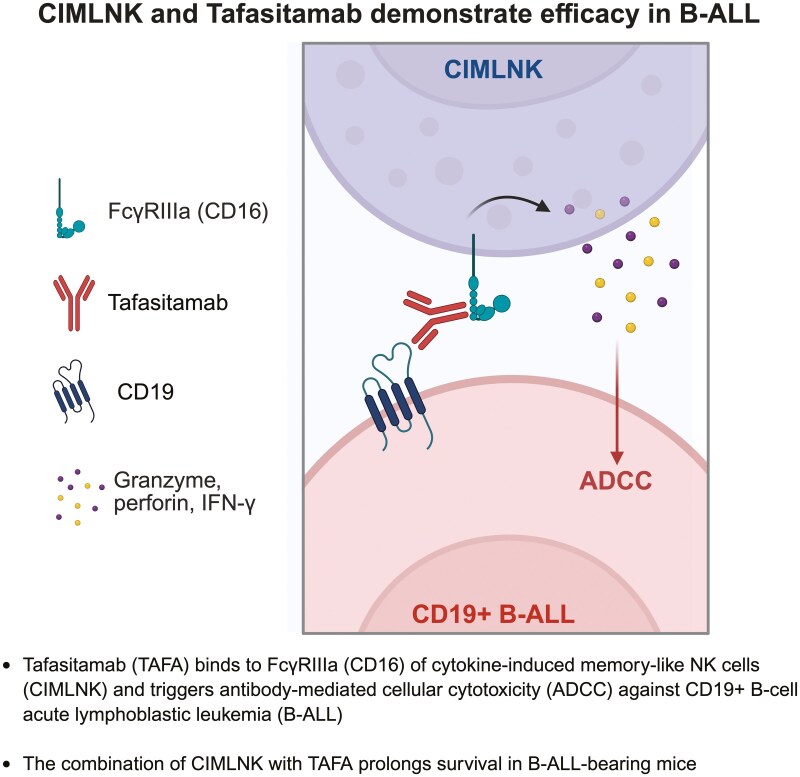

## Introduction

Natural killer (NK) cells are innate lymphoid cells that have gained significant interest in cancer immunotherapy due to their ability to exert anti-tumor effects in a non-MHC restricted manner and their lack of graft vs host disease induction after transplantation [1]. Recently, cytokine-induced memory-like NK cells (CIMLNK) have emerged as a promising therapeutic approach. CIMLNK cells are generated by overnight stimulation of NK cells with IL-12, IL-15, and IL-18 [2]. These cells exhibit distinct memory-like properties, enhanced anti-leukemic activity, and improved in vivo persistence compared to unstimulated NK cells [3–5]. CIMLNK therapy offers a safe cell-based treatment option due to its favorable toxicity profile including the absence of cytokine release syndrome (CRS) [4, 6–8]. Preclinical and clinical studies have demonstrated its efficacy in treating adult and pediatric acute myeloid leukemia (AML) particularly when administered following haploidentical hematopoietic stem cell transplantation (haplo-HSCT) [9–11]. However, the efficacy of CIMLNK against B-cell acute lymphoblastic leukemia (B-ALL) has been explored in a single study to date, which showed that B-ALL blasts of patients were more susceptible to lysis from CIMLNK derived from their haploidentical donors compared to unstimulated NK cells, highlighting the need for further investigation in this area [6].

B-ALL is the most common type of leukemia in the pediatric population with an overall survival rate of 85%. However, the prognosis in patients who relapse after hematopoietic cell transplantation (HCT) remains poor [12]. In adults, B-ALL poses an even greater challenge, with survival rates as low as 30% for patients over 65 years of age [13]. CD19 is a B cell antigen expressed during early stages of B-cell development and is present in almost all B-ALL [14–16]. Tafasitamab (TAFA) is a humanized anti-CD19 monoclonal antibody, with an Fc domain engineered for enhanced binding to FcγRIIIa. This modification increases its efficacy compared to its non-engineered CD19 IgG1 analog while inducing minimal CD19 internalization [17]. Although TAFA has been primarily used against diffuse large B-cell lymphoma (DLBCL), where it has demonstrated clinical efficacy, its application in B-ALL has been limited [18]. A recent clinical trial in adult patients with relapsed/refractory B-ALL showed that TAFA monotherapy was safe and induced short-term complete remissions [19]. The safety and efficacy of TAFA in pediatric B-ALL are currently being evaluated in an ongoing European clinical trial (NCT05366218). While TAFA can engage FcγRIIa on myeloid cells to facilitate antibody-derived cellular phagocytosis (ADCP) [20, 21], its antitumor activity is primarily mediated through binding to the high-affinity FcγRIIIa receptor on NK cells, thereby inducing antibody-derived cellular cytotoxicity (ADCC) [17, 22, 23]. CIMLNK have been shown to exhibit enhanced cytotoxicity and increased IFN-γ production following FcγRIIIa receptor engagement [24]. Therefore, we hypothesized that combining CIMLNK with TAFA would result in increased cytotoxic activity against B-ALL. Herein, we present both in vitro and in vivo evidence demonstrating that TAFA significantly enhances the anti-tumor efficacy of CIMLNK against B-ALL.

## Materials and methods

### Cell lines

For the in vitro experiments, RS4;11 (CRL-1873) ((t (4;11)) KMT2A-rearranged B-ALL), NALM6 (CRL-3273) (pre-B-ALL), and SUP-B15 (CRL-1929) ((t (9;22)) Ph+ B-ALL) were purchased from ATCC. For the in vivo experiments, NALM6-luc were kindly provided by Dr. Christian Capitini (University of Wisconsin School of Medicine and Public Health, Madison, WI). Cells were maintained by the addition of media every 2 days. Cell cultures were expanded for at least three passages before use in experiments. Mycoplasma testing was routinely performed. NALM6, NALM6-luc, RS4;11, NK and CIMLNK cells were all cultured in RPMI-1640 (Cytiva, Cat# SH30027.01) supplemented with 10% heat-inactivated fetal bovine serum (FBS) (GeminiBio, Cat# 100-500), HEPES (Gibco, Cat# 15630-080), Sodium Pyruvate (Cytiva, SH30239.01), and Penicillin/Streptomycin (P/S), (Gibco, 15140-122) at 37°C and 5% CO2. SUP-B15 were cultured in Iscove’s modified Dulbecco’s medium (IMDM) (Cytiva, Cat# SH30228.FS) supplemented with P/S, 20% FBS and 0.05 mM b-mercaptoethanol (Gibco, Cat# 21985-023). TAFA was purchased from SelleckChem (Cat# 1422527-84-1).

### NK cell isolation

Fresh peripheral blood mononuclear cells (PBMCs) were isolated from healthy donors by Ficoll density centrifugation. NK cells were then isolated and purified with the use of NK cell isolation kit (Miltenyi, Cat# 130-092-657) in the autoMACS NEO Pro Separator (Miltenyi). For the generation of CIMLNK, NK cells were incubated in media with recombinant human IL-12 (Miltenyi, Cat# 130-096-705) 10 ng/ml, IL-15 (Miltenyi, Cat# 130-095-760) 50 ng/ml and IL-18 (Creative Biomart, Cat# IL-18-536H) 50 ng/ml for 14-16 hours as previously described [10]. After the activation time, CIMLNK were washed to remove the cytokines before co-incubation with target cells or in vivo adoptive transfer.

### Flow cytotoxicity assay

ADCC with NKs and CIMLNKs as effectors was determined by flow cytometry. After labeling with human CD71-FITC (Miltenyi Cat#130-115-028) for 30 minutes on ice, 10,000 target cells (RS4;11, NALM6 or SUP-B15) were co-incubated in U-bottom 96-well plate with effectors cells at various E:T ratios and 1 μg/ml TAFA for 4 hours at 37°C. Specifically, for the TAFA titration assays, 1:1 E:T ratio was selected. Cells were then harvested, centrifuged, and resuspended with flow cytometry staining buffer (FACS). Propidium Iodide (PI) (Invitrogen Cat#00-6990-50) was added just before the analysis by flow cytometry (BD-LSR Fortessa). Live target cells were considered as CD71+PI- while the dead target cells were considered as CD71+PI+. Target cell cytolysis for each E:T ratio was calculated using the following formula: (%CD71+PI+ treated sample – %CD71+PI+ control sample), with control referred as the condition of target cells without effectors. Data analysis was performed using the FlowJo 10.9.0 software.

### Phenotypic analysis after cytotoxicity assay

To investigate the degree of CD16 utilization by TAFA in CIMLNK, cells were exited after cytotoxicity assay, resuspended in FACS buffer, and stained with anti-human CD16-APC (Miltenyi Biotec, Cat# 130-113-389) and anti-human CD56-APC-Vio® 770 (Miltenyi Biotec Cat# 130-114-548) for 30 minutes. Finally, cells were washed, resuspended in FACS buffer, and analyzed in BD-LSR Fortessa.

### Blocking assays

To assess that TAFA-mediated ADCC depended on TAFA binding to CD16, CIMLNK were stained with 3 μg/ml of unconjugated CD16 blocking antibody (StemCell, clone 3G8, Cat#60041.1) for 30 minutes in phosphate buffer saline (PBS) (Cytiva, Cat#SH30256.01) and then added directly on the 96-well plate containing CD71-labeled NALM6 at 1:1 E:T ratio +/- TAFA, followed by the 4 hours incubation in 37°C and 5% CO2 for a flow based cytotoxicity assay as described above.

### Degranulation and IFN-γ release assays

NK and CIMLNK cells were incubated with CD71-labeled NALM6 at 2.5:1 E/T ratio in 37°C and 5% CO2, in the presence of a human CD107a-APC antibody (BioLegend Cat# 328620). After one hour, Brefeldin A (Golgi Plug, BD Biosciences, Cat#555029) and Monensin (Golgi Stop; BD Biosciences, Cat#554724) was added. Following an additional 3-hour incubation, cells were centrifuged, resuspended in PBS, and stained with hCD56-PE antibody (BioLegend Cat# 362507) for 30 min on ice. Cells were then fixed for 30 minutes, permeabilized (Invitrogen, Cat#00-5523-00) and then stained with human IFN-γ-BV421 (BioLegend Cat# 502532) for 30 minutes on ice in the dark. Finally, cells were washed and resuspended on 200 μl of PBS followed by analysis on BD-LSR Fortessa.

### In vivo tumor model

8–12 weeks old female NOD.Cg-Prkdc^scid^ Il2rg^tm1Wjl^ Tg(IL15)1Sz/SzJ (NSG-IL15) mice were purchased from The Jackson Laboratory and bred at the University of Arizona Experimental Mouse Shared Resource. Female mice ages 8–12 weeks were randomized by weight and used for experiments. All mice had ad libitum access to food and water and were maintained on a 12-hour light–dark cycle in specific-pathogen free conditions. Mice were irradiated on day −1 with 150 cGy by RadSource X-ray irradiator. On day 0, following three washes with PBS, mice received 10,000 NALM6-luc resuspended in PBS via tail vein injection (IV). On day +1, 1 × 10^^6^ CIMLNK derived from 4 healthy human donors (*n* = 4) were injected IV in PBS. TAFA was administered intraperitoneally (IP) 1 mg/kg in PBS on day +1 and then every 2 days for a total of four doses. A separate group of NSG-IL15 mice received 1,000 NALM6-luc IV. Each mouse was given the appropriate medication or the corresponding vehicle. Mice were weighed twice weekly and monitored daily for survival. Animals losing more than 30% of starting weight for two consecutive weigh-ins or experiencing total hind-limb paralysis were euthanized.

### Bioluminescence imaging

Bioluminescence imaging (BLI) was performed weekly to assess the tumor burden. Briefly, after receiving a 200-μl IP injection of D-luciferin (15 mg/ml, GoldBio, Cat#Luck-1g), mice were anesthetized with 2% isoflurane, and then imaged using the Spectral Instruments Lago-X (Tucson, AZ, USA) system. BLI was quantified using the Aura imaging software and presented as photons/sec in a region of interest that includes the entire animal. BLI images were gathered following a 5-minute exposure. For visual representation, scales were adjusted to a radiance scale minimum of 1.7x 10^4^ and maximum of 5.5 × 10^7^ and saved as JPEG files.

### Peripheral blood engraftment

On day 8 and day 15, peripheral blood was collected from the tail vein of mice and processed to confirm the presence and assess the persistence of CIMLNK. After lysis of red blood cells (RBCs) at room temperature in 1× lysis buffer (BD biosciences, Cat# 555899), cells were washed and then resuspended in FACS buffer for subsequent flow cytometry staining. Anti-mouse CD45 Alexa Fluor 488 antibody (BioLegend Cat# 103122) was used to exclude mouse leukocytes from the analysis. The following antibodies were used to stain CIMLNK and confirm the absence of contamination from other cells: hCD56-PE antibody, (BioLegend Cat# 362507), hCD45 BV421 (BioLegend Cat# 304032), hCD3 PerCP/Cyanine5.5 (BioLegend Cat# 300430).

### Statistical analysis

All analyses were performed with the use of GraphPad Prism V10. ANOVA with Tukey’s posthoc analysis were performed to compare the groups among the different culture conditions unless stated otherwise. For the in vivo experiments, Kaplan Meier survival graphs were analyzed with the use of Log-rank Mantel-Cox test. BLI data were compared with a mixed linear mode using Tukey’s post-hoc analysis to assess the differences between the group. Significance was accepted at *P* < .05. The asterisks indicate increasing levels of significance with *<.05, **<.01, ***<.001, ****<.0001.

### Ethics statement

This study was approved and monitored by the University of Arizona Institutional Review Board for ethical conduct of research, in adherence with the standards of medical research involving humans as recommended by the Declaration of Helsinki. Peripheral blood was isolated from healthy participants (age 23–40 years old) that signed the written informed consent. All protocols and plans for the animal experiments were approved by The University of Arizona Institutional Animal Care and Use Committee (IACUC) (Protocol 14-517, 02/06/2020). The current manuscript is adherent with the ARRIVE guidelines.

## Results

### Combining TAFA with CIMLNK enhances cytotoxicity against B-ALL

**Figure 1.**
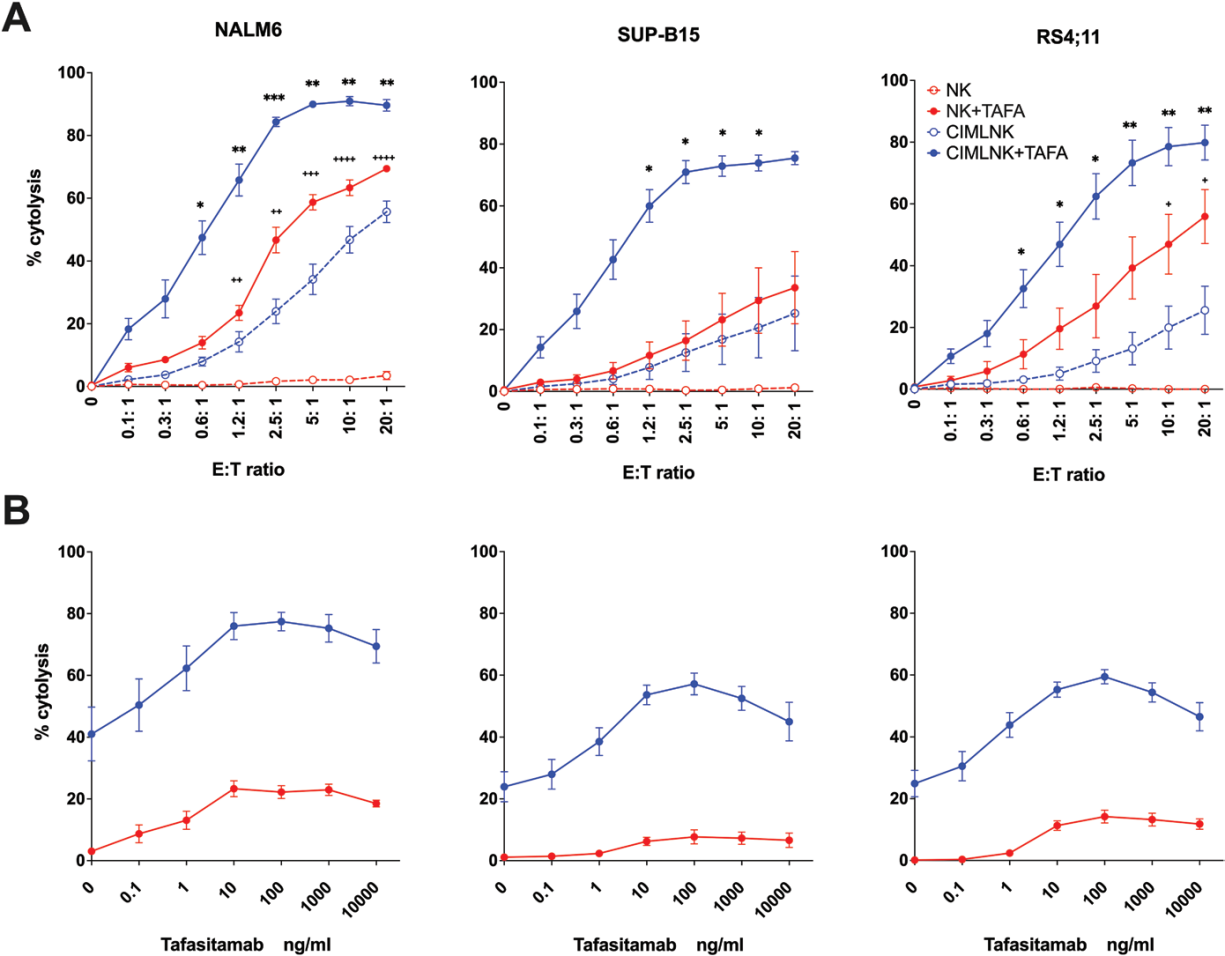
(A): CIMLNK with TAFA demonstrate enhanced cytotoxic capacity against B-ALL. CD71 pre-labeled target cells (NALM6 or SUP-B15 or RS4;11) were co-incubated with effectors (NK and CIMLNK) at various E: T ratios in the presence or absence of 1 μg/ml TAFA for four hours. Target cell specific cytolysis was calculated as the percentage of CD71+PI+. Summary data are the mean values +/- SEM from 4 different donors for SUP-B15 (n=4) and RS4;11 (n=4) and 5 different donors for NALM6 (n=5). Cytolysis differences between NK+TAFA and CIMLNK+TAFA were statistically significant between 1.2:1 and 20:1 E:T ratio for NALM6 and between 0.6:1 and 5:1 E:T ratio for SUP-B15. Statistically significant difference between CIMLNK and CIMLNK+TAFA for each E:T ratio depicted by * and between NK and NK+TAFA by ^+^; *P* * or ^+^ <.05, ** or ^++^<.01, *** or ^+++^ <.001, **** or ^++++^<.0001. (B): TAFA dose-dependently enhances ADCC in CIMLNK. CD71 pre-labeled target cells (NALM6 or SUP-B15 or RS4;11) were incubated with effectors (NK and CIMLNK) at 1:1 E:T ratio in the presence of different TAFA concentrations for 4 hours. Data points indicate mean values +/- S.E.M. of duplicate wells from 4 different donors (n=4). Statistically significant difference between CIMLNK with or without TAFA was achieved at TAFA concentrations of 10 ng/ml for NALM6 and 1 ng/ml for both SUP-B15 and RS4;11. For NK cells, the addition of TAFA led to a statistically significant increase at 1 ng/ml against NALM6 and at 10 ng/ml against RS4;11, while no statistically significant effect was not observed against SUP-B15.

We investigated whether the combination of CIMLNK and TAFA could enhance anti-tumor activity against CD19+ B-ALL cell lines in vitro. Three representative B-ALL subtypes were selected all of which are resistant to lysis by unstimulated NK cells (Fig. 1A). In cytotoxicity assays, we found that CIMLNK alone exerted significantly higher cytotoxicity compared to the unstimulated NK cells against NALM6 (*P* = .03) and showed non-significant increases against SUP-B15 (*P* = .33) and RS4;11 (*P* = .17). TAFA alone had no measurable cytotoxic effect on any of the cell lines (data not shown). However, the combination of TAFA and CIMLNK resulted in a significant enhancement in cytotoxicity across all B-ALL cell lines compared to CIMLNK monotherapy, achieving close to maximum cytolysis rates of 90.9% for NALM6 (*P* = .003), 78.5% for RS4;11 (*P* = .005) and 73.8% for SUP-B15 (*P* = .04) at 10:1 E:T ratio. Moreover, the presence of TAFA significantly enhanced CIMLNK-mediated cytotoxicity compared to unstimulated NK cells against NALM6 (*P* = .009) and SUP-B15 (*P* = .02), with a similar trend observed for RS4;11 (*P* = .08). Notably, CIMLNK + TAFA combination exhibited ADCC even at low E:T ratios in all cell lines tested (Fig. 1A). These results demonstrate that TAFA-triggered ADCC significantly potentiates CIMLNK-mediated lysis of B-ALL cells, underscoring its potential as a therapeutic strategy for resistant B-ALL subtypes.

### TAFA enhances CIMLNK-mediated ADCC in a dose-dependent manner

Previous studies have shown that TAFA enhances NK cell-mediated ADCC in a dose-dependent manner [22, 25]. To determine if this effect extends to CIMLNK, we performed a TAFA dose titration using either NK or CIMLNK as effectors against NALM6, RS4;11 and SUP-B15. Across all cell lines, TAFA-triggered ADCC was significantly enhanced compared to CIMLNK control cytotoxicity when TAFA concentration exceeded 1 ng/ml, except for NALM6 in which TAFA-induced ADCC reached significance at 10 ng/ml. Within the range of 10–1000 ng/ml, lysis rates were comparable across all three cell lines (Fig. 1B). In contrast, TAFA induced significant NK-mediated ADCC at 1 ng/ml against NALM6 and at 10 ng/ml against RS4;11 but had no significant effect against SUPB-15. These findings indicate that TAFA, when combined with CIMLNK, elicited a broadened response against all 3 B-ALL subtypes, demonstrating efficacy in settings where unstimulated NK cells show limited activity.

### CIMLNK cultured in combination with TAFA, and B-ALL exerts enhanced degranulation and IFN-γ secretion

**Figure 2.**
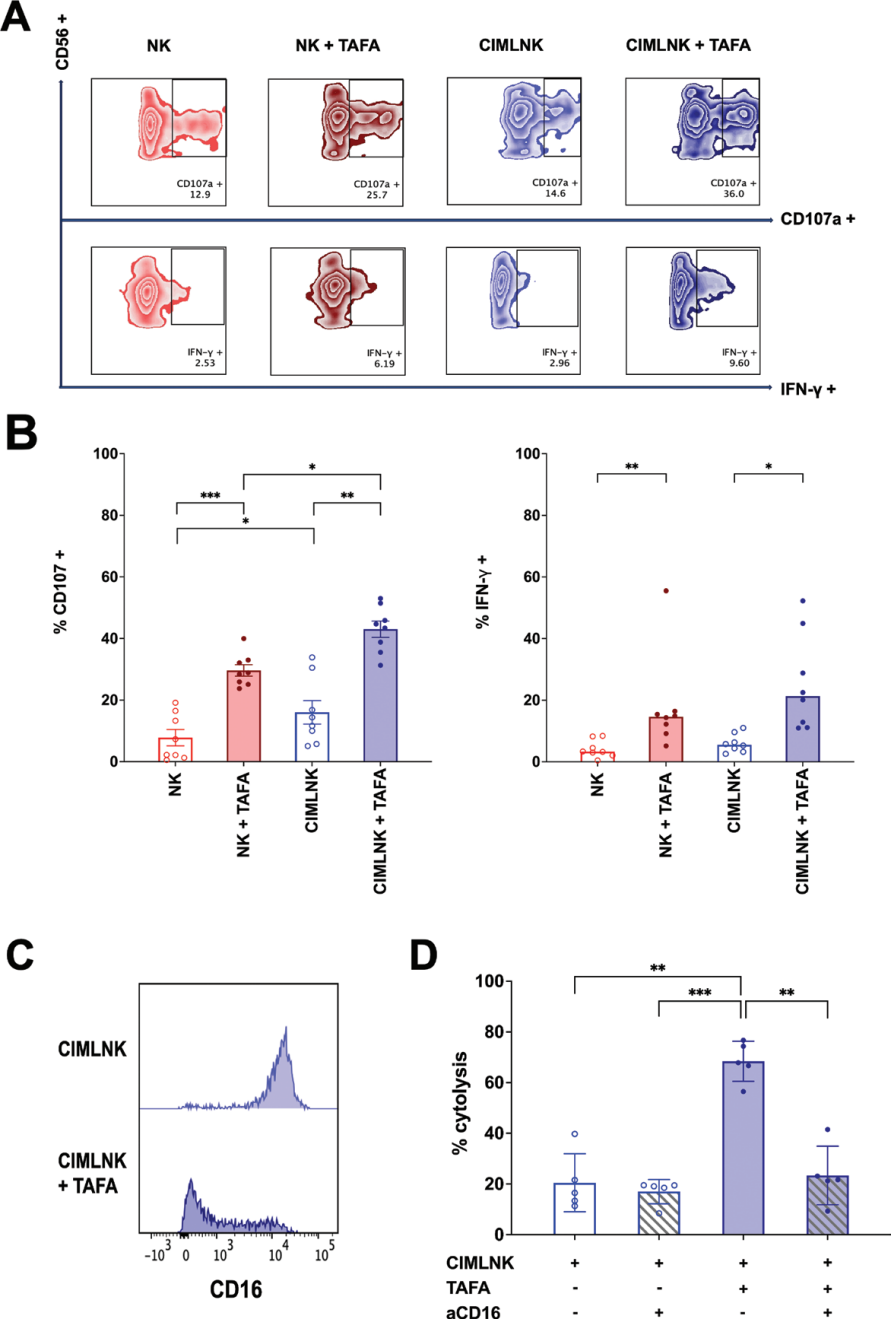
(A): Representative flow gating strategy depicting degranulation (CD107a) and IFN-γ release in CIMLNK cells (stained with CD56-PE), with or without TAFA, in one experiment. CD71 pre-labeled target cells (NALM6) were incubated with effectors (NK and CIMLNK) at 2.5:1 E:T ratio in the presence or absence of 1 μg/ml TAFA for 4 hours. (B): Summary degranulation and IFN-γ release data of NK or CIMLNK cells, with/without TAFA. For degranulation, data were analyzed using 2-way ANOVA with Tukey’s multiple comparisons test. Due to violation of the normality assumption in the NK + TAFA group, IFN-γ secretion data were analyzed using the non-parametric Friedman test. Data for degranulation are the mean values +/- SEM while IFN-γ values represent the medians from 8 independent experiments (*n* = 8). *P* *<.05, **<.01, ***<.001. (C): Histogram plots showing CD16 expression in CIMLNK cells (CD56+CD71-) after 4-hour incubation with or without TAFA. (D): TAFA-triggered ADCC is abolished with CD16 blockade. CIMLNK pre-labeled with 3 μg/ml of CD16 blocking antibody (aCD16) were co-incubated with CD71 pre-labeled NALM6 at 1:1 E:T ratio, with or without 1 μg/ml TAFA for 4 hours. Data points indicate mean values +/- S.E.M. of duplicate wells from five different experiments (*n* = 5). Data were analyzed using a one-way ANOVA with Tukey’s multiple comparison test. *P* *<.05, **<.01, ***<.001.

We next investigated whether adding TAFA to CIMLNK could enhance their mechanistic responses against NALM6. Specifically, we assessed degranulation, indicated by surface expression of CD107a and intracellular IFN-γ expression in both NK and CIMLNK upon stimulation with NALM6, with or without TAFA (Fig. 2A). CIMLNK alone showed significantly greater degranulation compared to unstimulated NK cells (16% vs 7.8%, *P* = .01) but had comparable IFN-γ secretion (5.5% vs 3.2%, *P* > 0.9) (Fig. 2B). The addition of TAFA significantly enhanced the degranulation capacity of both NK cells (29.6% vs 7.8%, *P* = 0.0002) and CIMLNK cells (43% vs 16%, *P* = .006). Similarly, TAFA potentiated the IFN-γ secretion in both NK cells (14.6%, *P* = .006) and CIMLNK cells (21.3%, *P* = .02). Comparing the TAFA-mediated responses between CIMNLNK and unstimulated NK cells, CIMLNK exhibited significantly greater degranulation in the presence of TAFA (*P* = .01) and a non-significant increase in IFN-γ secretion (*P* > .9). These findings demonstrate that while TAFA enhances the anti-tumor functions of both NK and CIMLNK, its effects are significantly more pronounced with CIMLNK, particularly in enhancing degranulation.

### CD16 blockade abrogates TAFA-mediated ADCC

Given that TAFA is designed to have enhanced affinity for CD16, we investigated whether the CIMLNK+TAFA cytotoxic effect depends on TAFA’s binding to CD16. First, we evaluated whether CD16 expression in CIMLNK is altered following coculture with TAFA and target cells (NALM6) during the cytotoxicity assay. Our analysis revealed a significant downregulation of CD16 in CIMLNK cells exposed to TAFA and target cells, suggesting that CD16 was actively engaged by CIMLNK cells (Fig. 2C). To further investigate, we conducted in vitro cytotoxicity assays with and without a CD16-specific blocking antibody. Our results showed that TAFA-induced ADCC was completely abolished in the presence of the CD16 blocking antibody (Fig. 2D). These findings confirm that CD16 ligation in CIMLNK is the critical mechanism underlying TAFA-triggered ADCC.

### CIMLNK+TAFA combination demonstrates superior antitumor effects and prolongs survival of leukemia-bearing mice

**Figure 3.**
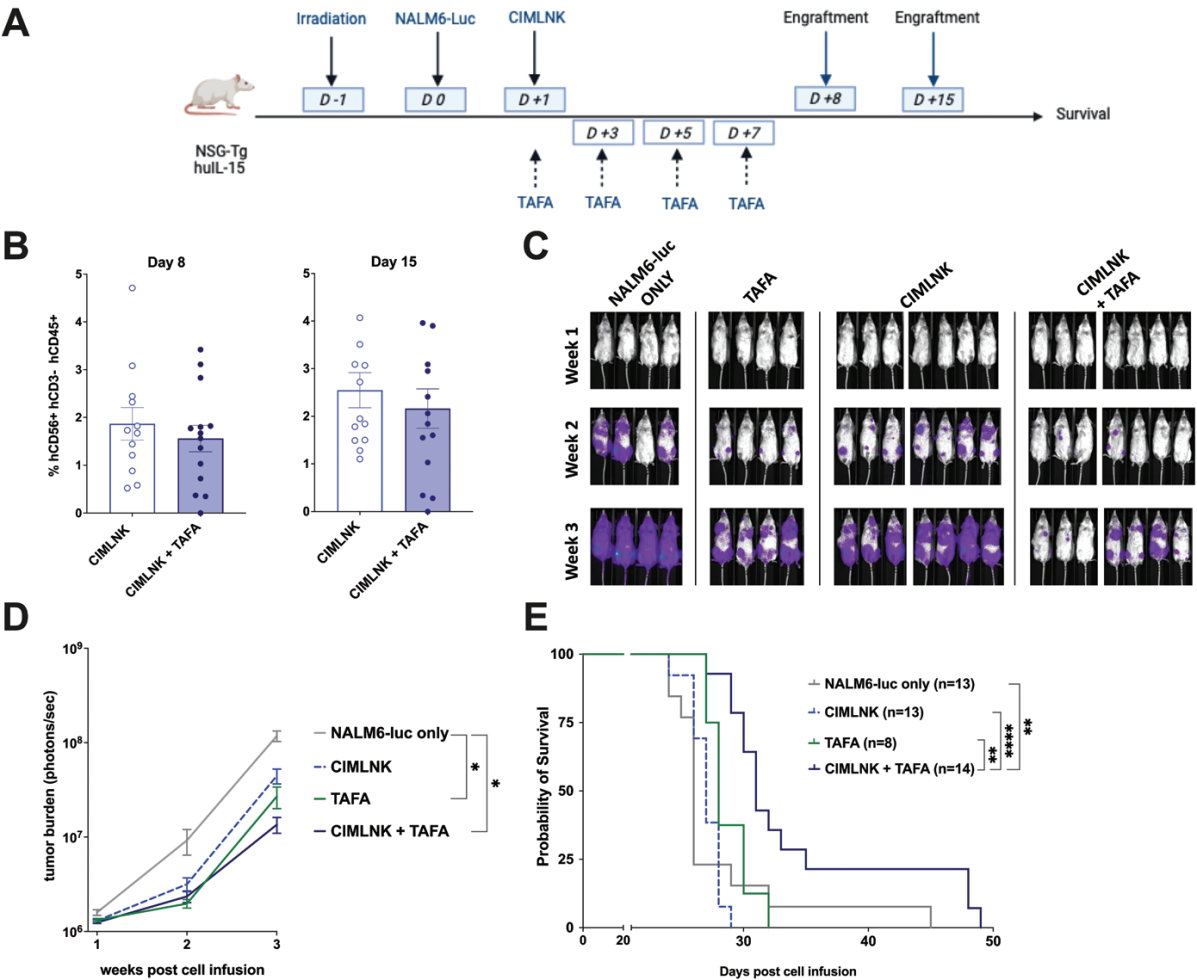
(A): Efficacy of CIMLNK +/- TAFA in vivo. Schema of the in vivo studies. Briefly, irradiated mice received 1 × 10^4^ Nalm6-luc cells IV on day 0 followed by 1 × 10^6^ CIMLNK cells on day +1. TAFA was administered at 1 mg/kg every other day IP from day +1 until day +7. Created in https://Biorender.com. (B): Summary BLI data of the tumor burden of each group monitored once weekly for 3 weeks after tumor inoculation. At week 3, data were analyzed using a mixed linear model with Tukey’s post-hoc test to assess the differences between the group. *P* *<.05, **<.01, ***<.001, ****<.0001. (C): Representative BLI images from one experiment at the indicated time points. (D): Human cell engraftment in mice. The number of human CIMLNK (hCD56+ hCD3- hCD45+) cells in the peripheral blood of mice was assessed on days +8 and +15. (E): Kaplan–Meier survival curve of mice receiving tumor only (*n* = 13), TAFA only (*n* = 8), CIMLNK only (*n* = 13), and CIMLNK + TAFA (*n* = 14). Data were pooled from three independent experiments. Comparison between groups was done using the log-rank test. *P* *<.05, **<.01, ***<.001, ****<.0001.

We investigated whether the TAFA-mediated ADCC could improve disease-free survival in immunocompromised leukemia-bearing mice. NSG-IL15 mice, which produce near-physiological levels of IL-15 to support CIMLNK persistence in vivo, were used for these studies [26]. Peripheral blood was isolated on days 8 and 15 post-treatment to assess CIMLNK persistence. CIMLNK were detectable at low levels (~2%) on both days, with no significant differences observed between the CIMLNK monotherapy and CIMLNK+TAFA combination groups (Fig. 3A, 3B). Using NALM6-luc as an in vivo xenogeneic leukemia model, NSG-IL15 mice were injected intravenously with leukemia cells and treated the following day with a single intravenous dose of CIMLNK and followed by intraperitoneal injections of TAFA. TAFA was administered every other day for a total of four doses. Tumor burden was monitored using BLI. Three weeks post-leukemia inoculation, both TAFA monotherapy and the combination of CIMLNK+TAFA restricted leukemia progression (*P* = .04 and *P* = .01, respectively), whereas CIMLNK monotherapy did not suppress BLI signal (*P* = .3) (Fig. 3C, 3D). Median survival of untreated mice was 26 days. CIMLNK and TAFA monotherapies extended survival modestly to 27 and 28 days, respectively, but only the CIMLNK+TAFA combination significantly prolonged survival to 31 days (*P* = .002) (Fig. 3E). To evaluate the magnitude of efficacy against this aggressive leukemia model, a separate cohort of mice was injected with 1,000 NALM6-luc. This group receiving one log less leukemia showed a median survival of 32 days and BLI signal at 3 weeks both comparable to mice treated with CIMLNK+TAFA combination (Supplementary Figs A–C). These findings highlight that the CIMLNK+TAFA combination significantly prolongs survival and exhibits antitumor effects in vivo, demonstrating its therapeutic potential against B-ALL in vivo.

## Discussion

CIMLNK, generated through brief stimulation of NK cells with the cytokines IL-12, IL-15, and IL-18 [2], offer a simple, novel and alternative form of cellular therapy with a low incidence of adverse events [4–6]. Cytokine-induced memory-like NK (CIMLNK) cells have been extensively studied in AML but have not demonstrated comparable efficacy in B-ALL. Therefore, in this study, we evaluated the efficacy of CIMLNK in combination with the anti-CD19 monoclonal antibody Tafasitamab (TAFA) as a novel immunotherapeutic approach against CD19+ B-ALL. Our findings demonstrate that the CIMLNK+TAFA combination significantly enhances anti-tumor activity in vitro and in vivo, enabling the targeted elimination of NK-cell-resistant CD19+B-ALL.

In contrast to the autologous cell collection required for CAR-T therapy following HCT, NK cells can be conveniently obtained from the original stem cell donor. Once collected, NK cells can be prepared and infused within 24 hours, unlike CAR-T cells, which require adequate patient T cell counts for collection and involve lengthy manufacturing times spanning several weeks. CIMLNK derived from haploidentical donors have been successfully utilized as a post-haplo-HSCT therapy in the treatment of AML and other myeloid neoplasms [5, 9, 10, 27]. However, there is limited data on CIMLNK activity against B-ALL, with only one study reporting their efficacy against patient-derived B-ALL cells [6]. A more recent advancement involves the development of CD19 CAR engineered CIMLNK, which have demonstrated preclinical effectiveness against CD19+ tumors including B-ALL [28, 29]. Despite their promise, CAR-based technologies are associated with significant financial burdens and manufacturing challenges, which may limit their widespread application especially in less developed countries [7, 30].

Unstimulated NK cells have been reported to exhibit limited cytotoxicity against B-ALL blasts primarily due to insufficient activation through MHC class I chain-related gene AB (MICA/B) [31]. Additionally, a recent clinical study revealed that NK cells in patients with B-ALL are not only reduced in number but also functionally impaired [32]. Further challenges associated with NK cell therapies include their short persistence and the need for a KIR-mismatched haploidentical donor. This mismatch minimizes NK cell inhibition by host-human leukocyte antigen (HLA)-I on leukemic blasts while preserving beneficial interactions between KIR and KIR-ligands [33, 34]. However, simultaneous stimulation with IL-12, IL-15, and IL-18 generates a differentiated NK cell population with potent antileukemia activity independent of KIR licensing status [24]. Notably, CIMLNK cells have been found to exert anti-tumor responses and enhanced IFN-γ production irrespective of inhibitory KIR interactions [11]. Ewen et al. demonstrated that KIR2DL2/L3, KIR2DL1, and KIR3DL1 downregulation following extended IL-12,15,18 pre-activation enhanced CD16-dependent ADCC by CIMLNK, despite concurrent CD16 downregulation post-stimulation [35]. Additionally, a previous study reported that TAFA dose-dependently enhanced NK cell ADCC against MLL-rearranged B-ALL, achieving up to 60% cytolysis against RS4:11 and improving survival of leukemia-bearing NOG mice. Notably, ADCC and survival of mice were significantly enhanced with the addition of a pan-MHC inhibitor, which blocks all the interactions between inhibitory KIRs and HLAs, highlighting the importance of selecting KIR-mismatch donors for NK-mediated ADCC therapies [25]. Building on these insights, we hypothesized that combining CIMLNK with TAFA represents a rational and promising immunotherapeutic strategy against B-ALL.

We observed that combining TAFA with CIMLNK resulted in a significantly enhanced cytotoxicity against CD19+ B-ALL. Specifically, CIMLNK exerted significantly higher tumor cell lysis in the presence of TAFA compared to CIMLNK monotherapy, with this effect being consistent across all cell lines tested. These results suggest that TAFA-induced ADCC plays a key role in the anti-tumor activity of CIMLNK. Moreover, except for RS4;11, CIMLNK cells exhibited significantly higher TAFA- triggered ADCC compared to unstimulated NK cells. Notably, CIMLNK monotherapy showed significantly higher cytotoxicity against NALM6 but not against SUP-B15 or RS4;11, which represent more aggressive subtypes of B-ALL associated with worse clinical outcomes [36, 37]. Therefore, the combination of CIMLNK and TAFA demonstrate enhanced efficacy against B-ALL.

A previous study indicated that TAFA could trigger dose-dependent ADCC against both pediatric and adult patient-derived ALL blasts with high HLA class I expression when combined with autologous patient-derived NK cells. Interestingly, this ADCC was independent of the FcγRIII-V/F allotype [22]. Similarly, our findings demonstrate that TAFA is active at very low concentrations triggering significant ADCC in CIMLNK even at 1 ng/ml, which is within the achievable and sustained serum levels in patients [19]. Importantly, TAFA-induced lysis with CIMLNK was consistent within the range of 10–1000 ng/ml, with this pattern observed across all cell lines. NK-cell mediated ADCC occurs through the ligation of CD16 (FcγRIIIa) by the Fc portion of the monoclonal antibody [38]. Since CIMLNK also express CD16, we investigated whether TAFA-induced ADCC in CIMLNK was dependent on TAFA binding to CD16. Our results show that TAFA-mediated ADCC of CIMLNK is completely abolished when a CD16 blocking antibody is used. Therefore, CD16 is a critical component in the enhanced cytotoxic capacity of CIMLNK with TAFA.

CIMLNK are known to exert superior cytotoxic responses compared to unstimulated NK cells [2, 4]. Our findings demonstrated that when exposed to CD19+B-ALL, CIMLNK showed increased degranulation compared to unstimulated NK cells, although IFN-γ expression was similar between the two groups. This contrasts with the observed boost in IFN-γ secretion by CIMLNK against AML cell lines or patient samples [2, 4, 39]. However, when TAFA was added, both degranulation and IFN-γ expression were upregulated in CIMLNK and NK cells, with a more pronounced increase in degranulation observed in CIMLNK cells. Previous studies have shown that CD16 ligation with antibodies primes NK cells to secrete more IFN-γ [40]. Interestingly, we found that CIMLNK degranulated significantly more than unstimulated NK cells in the presence of TAFA, which correlated with the enhanced ADCC observed at this E:T ratio in our cytotoxicity assays. This finding aligns with previous reports that degranulation is closely correlated with NK-mediated ADCC [41].

CIMLNK demonstrate superior persistence in vivo compared to unstimulated NK cells [6, 11]. Indeed, adoptively transferred CIMLNK can persist in patients for at least 2 months [5, 39]. Traditionally, repeated IL- 2 injections have been used to support CIMLNK persistence in vivo in mice [11]. However, Tanzi et al. employed a different in vitro protocol, utilizing both IL-2 and IL-15 to support CIMLNK maintenance after IL-12, IL-15, and IL-18 stimulation before adoptive transfer into NSG mice. They reported that CIMLNK remained detectable at low levels in the blood of NSG mice for 19 days post-injection [6]. Similarly, we observed that CIMLNK were still detectable in the blood of NSG-IL15 mice 2 weeks after a single injection, albeit at low percentages (~2%). In our survival studies, CIMLNK monotherapy was largely ineffective against NALM6-luc. Interestingly, while TAFA alone did not induce direct cytotoxicity in our in vitro assays, it reduced the tumor burden in mice as observed through BLI. In a sub-analysis, we performed Annexin-PI assays to assess whether TAFA could induce direct cytotoxicity against B-ALL cell lines. However, we found no significant changes in the percentages of apoptotic or- dead cells after 24-hour coculture of target cells with increasing concentrations of TAFA (data not shown). Given that NSG mice lack NK cells and T cells [42], we hypothesize that the residual mouse macrophages may have mediated TAFA’s effects through antibody derived cellular phagocytosis (ADCP). In fact, a previous report has indicated that the remaining murine macrophages in NSG mice were able to respond to Toll-like receptor agonists and control a human patient-derived xenograft melanoma tumor [43]. A recent study showed that TAFA monotherapy, administered weekly, was able to prolong the survival of NSG mice bearing t (17;19) BCP-ALL [21]. Additionally, TAFA has been found to exhibit enhanced affinity not only for human but also for mouse Fc receptors [17]. However, in our in vivo experiment, TAFA administration alone did not improve survival of mice bearing NALM6-luc cells. Importantly, the combination of CIMLNK+TAFA was the only treatment regimen that significantly prolonged the survival of mice. To further assess treatment efficacy, we injected a separate group of mice with only 1,000 NALM6-luc cells. Interestingly, the CIMLNK+TAFA combination group demonstrated comparable tumor burden at week 3 and survival rates similar to the NALM6-luc untreated group receiving 1 log less leukemia cells. Thus, the CIMLNK+TAFA treatment resulted in a ~10-fold reduction in leukemia burden. These findings indicate that CIMLNK require TAFA to effectively exert their anti-tumor effect in vivo against B-ALL.

Our study has several limitations. First, the experiments were conducted using CIMLNK generated from consented healthy human donors, and we did not characterize donor-specific KIR expression or Fc polymorphisms. As such, it remains to be determined whether particular donor profiles are associated with enhanced ADCC activity. Additionally, validation and extension of our findings in primary B-ALL patient samples will be important in future studies.

In conclusion, we demonstrated that CIMLNK acted cooperatively with TAFA to effectively lyse CD19+ B-ALL both in vitro and in vivo. Our data highlight that the combination of CIMLNK and TAFA is a promising immunotherapy strategy against B-ALL. Given that CIMLNK derived from either the original matched sibling or haploidentical family donor can be readily available and that TAFA is actively being tested in pediatric B-ALL, the CIMLNK+TAFA combination could serve a viable alternative approach for patients with relapsed CD19+ B-ALL.

## Supplementary Material

ltaf025_suppl_Supplementary_Material

## Data Availability

The data supporting the findings of this study are available from the corresponding author upon reasonable request.

## References

[1] Liu S , Galat V, Galat Y et al NK cell-based cancer immunotherapy: from basic biology to clinical development. J Hematol Oncol 2021; 14(1):7. https://doi.org/10.1186/s13045-020-01014-w 33407739 PMC7788999

[2] Romee R , Schneider SE, Leong JW et al Cytokine activation induces human memory-like NK cells. Blood 2012; 120(24):4751–60. https://doi.org/10.1182/blood-2012-04-419283 22983442 PMC3520618

[3] Gang M , Wong P, Berrien-Elliott MM et al Memory-like natural killer cells for cancer immunotherapy. Semin Hematol 2020; 57(4):185–93. https://doi.org/10.1053/j.seminhematol.2020.11.003 33256911 PMC7810422

[4] Berrien-Elliott MM , Cashen AF, Cubitt CC et al Multidimensional analyses of donor memory-like NK cells reveal new associations with response after adoptive immunotherapy for leukemia. Cancer Discov 2020; 10(12):1854–71. https://doi.org/10.1158/2159-8290.CD-20-0312 32826231 PMC7710923

[5] Berrien-Elliott MM , Foltz JA, Russler-Germain DA et al Hematopoietic cell transplantation donor-derived memory-like NK cells functionally persist after transfer into patients with leukemia. Sci Transl Med 2022; 14(633):eabm1375. https://doi.org/10.1126/scitranslmed.abm1375 35196021 PMC9210521

[6] Tanzi M , Consonni M, Falco M et al Cytokine-Induced Memory-Like NK Cells with high reactivity against acute leukemia blasts and solid tumor cells suitable for adoptive immunotherapy approaches. Cancers (Basel) 2021; 13(7):1577. https://doi.org/10.3390/cancers13071577 33808051 PMC8036252

[7] Santomasso B , Bachier C, Westin J et al The other side of CAR T-Cell therapy: Cytokine Release Syndrome, neurologic toxicity, and financial burden. Am Soc Clin Oncol Educ Book 2019; 39:433–44. https://doi.org/10.1200/EDBK_238691 31099694

[8] Hüber CM , Doisne JM, Colucci F. IL-12/15/18-preactivated NK cells suppress GvHD in a mouse model of mismatched hematopoietic cell transplantation. Eur J Immunol 2015; 45(6):1727–35. https://doi.org/10.1002/eji.201445200 25778912 PMC4687420

[9] Bednarski JJ , Zimmerman C, Berrien-Elliott MM et al Donor memory-like NK cells persist and induce remissions in pediatric patients with relapsed AML after transplant. Blood 2022; 139(11):1670–83. https://doi.org/10.1182/blood.2021013972 34871371 PMC8931511

[10] Shapiro RM , Birch GC, Hu G et al Expansion, persistence, and efficacy of donor memory-like NK cells infused for posttransplant relapse. J Clin Invest 2022; 132(11):e154334. https://doi.org/10.1172/JCI154334 35349491 PMC9151697

[11] Romee R , Rosario M, Berrien-Elliott MM et al Cytokine-induced memory-like natural killer cells exhibit enhanced responses against myeloid leukemia. Sci Transl Med 2016; 8(357):357ra123. https://doi.org/10.1126/scitranslmed.aaf2341 PMC5436500 27655849

[12] Hunger SP , Raetz EA. How I treat relapsed acute lymphoblastic leukemia in the pediatric population. Blood 2020; 136(16):1803–12. https://doi.org/10.1182/blood.2019004043 32589723

[13] Sasaki K , Jabbour E, Short NJ et al Acute lymphoblastic leukemia: a population-based study of outcome in the United States based on the surveillance, epidemiology, and end results (SEER) database, 1980-2017. Am J Hematol 2021; 96(6):650–8. https://doi.org/10.1002/ajh.26156 33709456 PMC9517941

[14] Liedtke M , Cleary ML. Therapeutic targeting of MLL. Blood 2009; 113(24):6061–8. https://doi.org/10.1182/blood-2008-12-197061 19289854 PMC2699228

[15] Scheuermann RH , Racila E. CD19 antigen in leukemia and lymphoma diagnosis and immunotherapy. Leukemia Lymphoma 1995; 18(5-6):385–97. https://doi.org/10.3109/10428199509059636 8528044

[16] Rafiq S , Cheney C, Mo X et al XmAb-5574 antibody demonstrates superior antibody-dependent cellular cytotoxicity as compared with CD52- and CD20-targeted antibodies in adult acute lymphoblastic leukemia cells. Leukemia 2012; 26(7):1720–2. https://doi.org/10.1038/leu.2012.40 22333878 PMC3676652

[17] Horton HM , Bernett MJ, Pong E et al Potent in vitro and in vivo activity of an Fc-engineered anti-CD19 monoclonal antibody against lymphoma and leukemia. Cancer Res 2008; 68(19):8049–57. https://doi.org/10.1158/0008-5472.CAN-08-2268 18829563

[18] Duell J , Abrisqueta P, Andre M et al Tafasitamab for patients with relapsed or refractory diffuse large B-cell lymphoma: final 5-year efficacy and safety findings in the phase II L-MIND study. Haematologica 2024; 109(2):553–66. https://doi.org/10.3324/haematol.2023.283480 37646664 PMC10828760

[19] Klisovic RB , Leung WH, Brugger W et al A phase 2a, single-arm, open-label study of tafasitamab, a humanized, Fc-modified, anti-CD19 antibody, in patients with relapsed/refractory B-precursor cell acute lymphoblastic leukemia. Cancer 2021; 127(22):4190–7. https://doi.org/10.1002/cncr.33796 34343354 PMC9292493

[20] Gehlert CL , Rahmati P, Boje AS et al Dual Fc optimization to increase the cytotoxic activity of a CD19-targeting antibody. Front Immunol 2022; 13:957874. https://doi.org/10.3389/fimmu.2022.957874 36119088 PMC9471254

[21] Vogiatzi F , Heymann J, Müller K et al Venetoclax enhances the efficacy of therapeutic antibodies in B-cell malignancies by augmenting tumor cell phagocytosis. Blood Adv 2022; 6(16):4847–58. https://doi.org/10.1182/bloodadvances.2022007364 35820018 PMC9631674

[22] Kellner C , Zhukovsky EA, Pötzke A et al The Fc-engineered CD19 antibody MOR208 (XmAb5574) induces natural killer cell-mediated lysis of acute lymphoblastic leukemia cells from pediatric and adult patients. Leukemia 2013; 27(7):1595–8. https://doi.org/10.1038/leu.2012.373 23277329

[23] Coënon L , Villalba M. From CD16a biology to antibody-dependent cell-mediated cytotoxicity improvement. Front Immunol 2022; 13:913215. https://doi.org/10.3389/fimmu.2022.913215 35720368 PMC9203678

[24] Wagner JA , Berrien-Elliott MM, Rosario M et al Cytokine-induced memory-like differentiation enhances unlicensed natural killer cell antileukemia and FcγRIIIa-triggered responses. Biol Blood Marrow Transplant 2017; 23(3):398–404. https://doi.org/10.1016/j.bbmt.2016.11.018 27894857 PMC5408734

[25] Chan WK , Kung Sutherland M, Li Y et al Antibody-dependent cell-mediated cytotoxicity overcomes NK cell resistance in MLL-rearranged leukemia expressing inhibitory KIR ligands but not activating ligands. Clin Cancer Res 2012; 18(22):6296–305. https://doi.org/10.1158/1078-0432.CCR-12-0668 23014531 PMC3500445

[26] Aryee KE , Burzenski LM, Yao L-C et al Enhanced development of functional human NK cells in NOD-scid-IL2rg(null) mice expressing human IL15. FASEB J 2022; 36(9):e22476. https://doi.org/10.1096/fj.202200045R 35959876 PMC9383543

[27] Foltz JA , Berrien-Elliott MM, Neal C et al Cytokine-induced memory-like (ML) NK cells persist for > 2 months following adoptive transfer into leukemia patients with a MHC-compatible hematopoietic cell transplant (HCT). Blood 2019; 134(Supplement_1):1954–1954. https://doi.org/10.1182/blood-2019-126004

[28] He B , Mai Q, Pang Y et al Cytokines induced memory-like NK cells engineered to express CD19 CAR exhibit enhanced responses against B cell malignancies. Front Immunol 2023; 14:1130442. https://doi.org/10.3389/fimmu.2023.1130442 37207215 PMC10191231

[29] Gang M , Marin ND, Wong P et al CAR-modified memory-like NK cells exhibit potent responses to NK-resistant lymphomas. Blood 2020; 136(20):2308–18. https://doi.org/10.1182/blood.2020006619 32614951 PMC7702478

[30] Litvinova Y , Merkur S, Allin S et al Availability and financing of CAR-T cell therapies: a cross-country comparative analysis. Health Policy 2024; 149:105153. https://doi.org/10.1016/j.healthpol.2024.105153 39270403

[31] Romanski A , Bug G, Becker S et al Mechanisms of resistance to natural killer cell-mediated cytotoxicity in acute lymphoblastic leukemia. Exp Hematol 2005; 33(3):344–52. https://doi.org/10.1016/j.exphem.2004.11.006 15730858

[32] Duault C , Kumar A, Taghi Khani A et al Activated natural killer cells predict poor clinical prognosis in high-risk B- and T-cell acute lymphoblastic leukemia. Blood 2021; 138(16):1465–80. https://doi.org/10.1182/blood.2020009871 34077953 PMC8532198

[33] Shaffer BC , Hsu KC. How important is NK alloreactivity and KIR in allogeneic transplantation? Best Pract Res Clin Haematol 2016; 29(4):351–8. https://doi.org/10.1016/j.beha.2016.10.010 27890259 PMC5896016

[34] Page A , Chuvin N, Valladeau-Guilemond J et al Development of NK cell-based cancer immunotherapies through receptor engineering. Cell Mol Immunol 2024; 21(4):315–31. https://doi.org/10.1038/s41423-024-01145-x 38443448 PMC10978891

[35] Ewen EM , Pahl JHW, Miller M et al KIR downregulation by IL-12/15/18 unleashes human NK cells from KIR/HLA-I inhibition and enhances killing of tumor cells. Eur J Immunol 2018; 48(2):355–65. https://doi.org/10.1002/eji.201747128 29105756

[36] Qiu KY , Zhou D-H, Liao X-Y et al Prognostic value and outcome for acute lymphocytic leukemia in children with MLL rearrangement: a case-control study. BMC Cancer 2022; 22(1):1257. https://doi.org/10.1186/s12885-022-10378-w 36461002 PMC9719147

[37] Lejman M , Chałupnik A, Chilimoniuk Z et al Genetic biomarkers and their clinical implications in B-cell acute lymphoblastic leukemia in children. Int J Mol Sci 2022; 23(5):2755. https://doi.org/10.3390/ijms23052755 35269896 PMC8911213

[38] Wu J , Mishra HK, Walcheck B. Role of ADAM17 as a regulatory checkpoint of CD16A in NK cells and as a potential target for cancer immunotherapy. J Leukoc Biol 2019; 105(6):1297–303. https://doi.org/10.1002/JLB.2MR1218-501R 30786043 PMC6792391

[39] Foltz JA , Tran J, Wong P et al Cytokines drive the formation of memory-like NK cell subsets via epigenetic rewiring and transcriptional regulation. Sci Immunol 2024; 9(96):eadk4893. https://doi.org/10.1126/sciimmunol.adk4893 38941480 PMC12329699

[40] Capuano C , Pighi C, Molfetta R et al Obinutuzumab-mediated high-affinity ligation of FcγRIIIA/CD16 primes NK cells for IFNγ production. Oncoimmunology 2017; 6(3):e1290037. https://doi.org/10.1080/2162402X.2017.1290037 28405525 PMC5384385

[41] Wang Z , Guan D, Wang S et al Glycolysis and oxidative phosphorylation play critical roles in natural killer cell receptor-mediated natural killer cell functions. Front Immunol 2020; 11:202. https://doi.org/10.3389/fimmu.2020.00202 32153568 PMC7045049

[42] Shultz LD , Lyons BL, Burzenski LM et al Human lymphoid and myeloid cell development in NOD/LtSz-scid IL2Rγnull mice engrafted with mobilized human hemopoietic stem cells 12. J Immunol 2005; 174(10):6477–89. https://doi.org/10.4049/jimmunol.174.10.6477 15879151

[43] Aryee KE , Shultz LD, Burzenski LM et al NOD-SCID IL2rγnull mice lacking TLR4 support human immune system development and the study of human-specific innate immunity. J Leukoc Biol 2023; 113(5):418–33. https://doi.org/10.1093/jleuko/qiac020 36801998 PMC12184879

