# Supplementary material for: Cytokine-induced memory-like NK cells combined with Tafasitamab demonstrate efficacy against B-cell acute lymphoblastic leukemia
Source: Immunother Adv. 2025 Jul 16;5(1):ltaf025. doi: 10.1093/immadv/ltaf025 (PMC12264592; doi:10.1093/immadv/ltaf025)
Supplement: ltaf025_suppl_Supplementary_Material [file ltaf025_suppl_supplementary_material.docx]

**Supplemental figures**

**
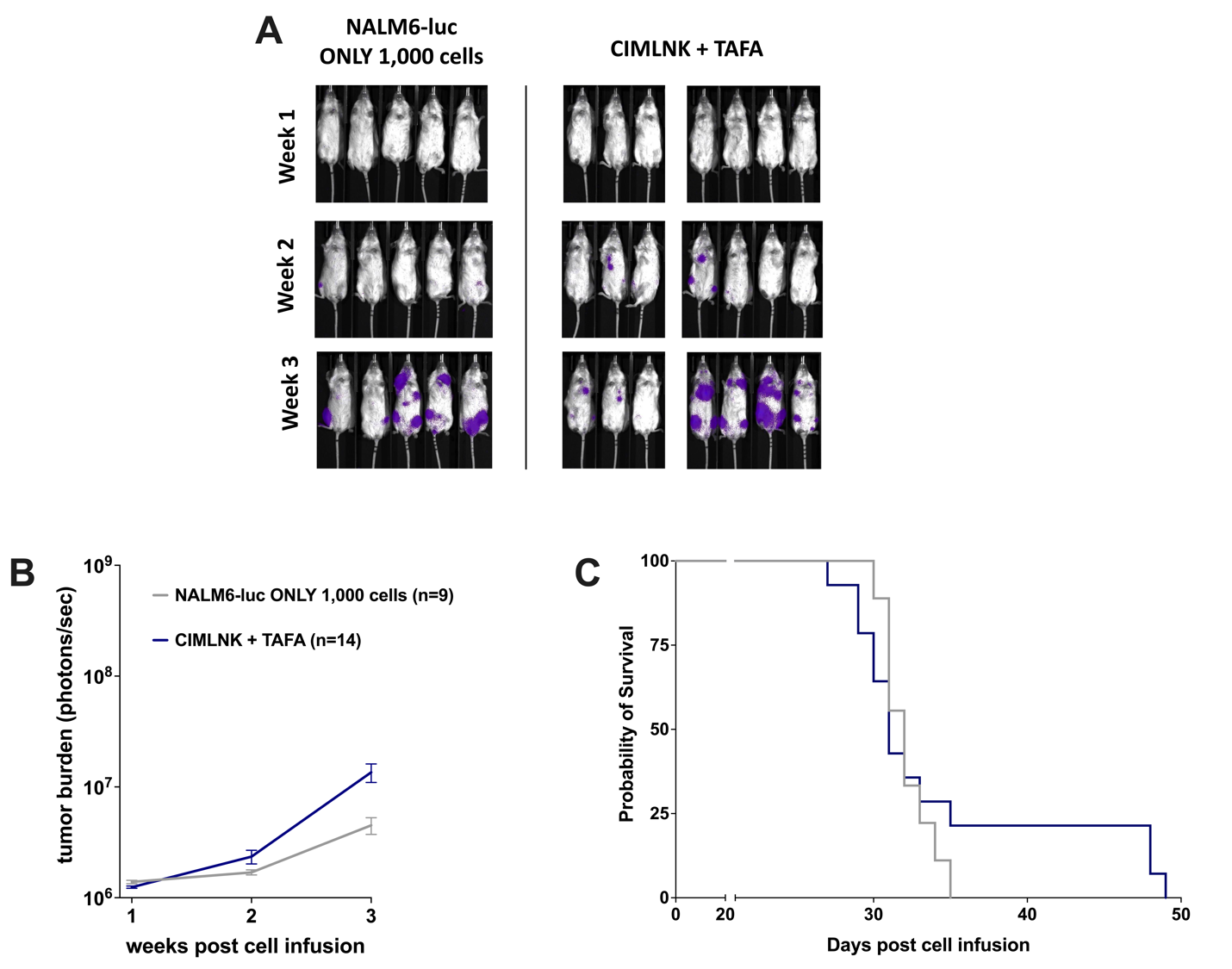
**

(A): Representative BLI images from one experiment at the indicated time points comparing tumor burden in mice that received CIMLNK+TAFA+ 10,000 NALM6-luc cells vs 1,000 NALM6-luc cells only.

(B): Summary BLI data of the tumor burden of each group monitored once weekly for 3 weeks after tumor inoculation. At week 3, data were analyzed using a mixed linear model with Tukey’s post-hoc test to assess the differences between the group.

(C): Kaplan-Meier survival curve of mice receiving CIMLNK + TAFA (blue line) (n=14), tumor only – 1,000 cells (grey dotted line) (n=9). P *<0.05, **<0.01, ***<0.001, ****<0.0001.
